# Allorecognition Triggers Autophagy and Subsequent Necrosis in the Cnidarian *Hydractinia symbiolongicarpus*

**DOI:** 10.1371/journal.pone.0048914

**Published:** 2012-11-08

**Authors:** Leo W. Buss, Christopher Anderson, Erica Westerman, Chad Kritzberger, Monita Poudyal, Maria A. Moreno, Fadi G. Lakkis

**Affiliations:** 1 Department of Ecology and Evolutionary Biology, Yale University, New Haven, Connecticut, United States of America; 2 Department of Molecular, Cell and Developmental Biology, Yale University, New Haven, Connecticut, United States of America; 3 Thomas E. Starzl Transplantation Institute, Departments of Surgery, Immunology, and Medicine, University of Pittsburgh School of Medicine, Pittsburgh, Pennsylvania, United States of America; Ecole Normale Supérieure de Lyon, France

## Abstract

Transitory fusion is an allorecognition phenotype displayed by the colonial hydroid *Hydractinia symbiolongicarpus* when interacting colonies share some, but not all, loci within the allorecognition gene complex (ARC). The phenotype is characterized by an initial fusion followed by subsequent cell death resulting in separation of the two incompatible colonies. We here characterize this cell death process using scanning electron microscopy (SEM), transmission electron microscopy (TEM), and continuous *in vivo* digital microscopy. These techniques reveal widespread autophagy and subsequent necrosis in both colony and grafted polyp assays. Terminal deoxynucleotidyl transferase dUTP nick end labeling (TUNEL) assays and ultrastructural observations revealed no evidence of apoptosis. Pharmacological inhibition of autophagy using 3-methyladenine (3-MA) completely suppressed transitory fusion *in vivo* in colony assays. Rapamycin did not have a significant effect in the same assays. These results establish the hydroid allorecognition system as a novel model for the study of cell death.

## Introduction

Allorecognition phenomena are widely appreciated in the biomedical contexts of pregnancy and graft rejection, where natural maternal fetal interactions or surgical procedures lead to cell-cell contact between genetically distinct individuals. The adaptive immunoregulatory orchestra is elicited in response to foreign grafts [1] and selectively suppressed during pregnancy [2]. The capacity for allorecognition is not restricted to mammals. Encrusting colonial marine organisms often encounter conspecifics by the simple expedient of growing into contact with them [3]. Such interactions result not only in recognition, but the deployment of taxon-specific defenses.

Within colonial cnidarians, natural allorecognition interactions result in either fusion or rejection [4]–[7]. Fusion is characterized by adhesion of epidermal cells, followed by the establishment of gastrovascular endodermal continuity. Fusion typically results in the formation of a permanent genetic chimera. Rejection is characterized by a failure of ectodermal tissues to adhere and induction of a specialized tissue to which nematocytes amass [7]–[10]. Nematocysts subsequently discharge to effect local tissue destruction. Many solitary cnidarians likewise reject conspecifics, while tolerating closely related individuals [11]–[17].

Rejection mechanisms amongst cnidarians are invariably based on the discharge of nematocysts. The type of nematocyst employed and the type of tissue induced to deploy them vary widely: some hydroids employ specialized stolon tips [7], [8], called hyperplastic stolons, for aggression, some sea anemones utilize specialized body wall swellings called acrorhagi [11]–[13], [18] or modified catch tentacles [14], [15], [17], [19] in defense, while corals may use either mesenterial filaments [20]–[22] or inducible sweeper tentacles [23], [24] to engage conspecifics. The diversity of different cnidarian responses led us, long ago, to predict that the nematocyst-based effector systems of cnidarians were late evolutionary additions to an earlier recognition and effector response [8]. Recent progress in understanding the genetics of allorecognition in the colonial hydroid *Hydractinia symbiolongicarpus* provides an opportunity to explore this suggestion.

In *Hydractinia*, encounters between colonies may result not only in fusion or rejection, but also in an intermediate phenotype known as transitory fusion [4], [5], [25], [26]. First recognized in the 1950’s, transitory fusion displays the same phenomenology as fusion at the outset, with the important difference that cells in the fusion zone soon die and the colonies separate without nematocyst involvement. The transitory fusion phenotype is under the genetic control of the *Hydractinia* allorecognition gene complex (ARC) [26], [27]. The complex was first identified using classical breeding techniques as a single chromosomal interval [28] comprised of at least two linked Mendelian loci [27]. Positional cloning of these two loci showed the two genes, *alr1* and *alr2*, to be membrane-spanning molecules with extracellular immunoglobulin superfamily-like (IgSF-like) domains [29], [30]. Both molecules are extraordinarily polymorphic and display hallmarks of positive selection. The ARC chromosomal interval is populated not only by these two loci, but also by a large number of additional IgSF-like genes and pseudogenes [30]. Substantial evidence suggests that diversity at these loci is generated in some measure by sequence donation between known allodeterminants and ARC-encoded pseudogenes and between IgSF-like ARC loci [30], [31]. Within congenic lines, the transitory fusion phenotype arises when colonies share alleles at one *alr* locus and not the other [26], [27].

The availability of defined genetic lines provides an opportunity to characterize the transitory fusion phenotype in detail. We here describe the process based on SEM, TEM, *in vivo* continuous, stage-mounted, time-lapse digital microscopy and experimental manipulation of cell death pathways. We find no evidence for a role of apoptosis in the transitory fusion response. Autophagy was found to be widespread in contact zones between colonies and in allogeneic polyp grafts. Pharmacological inhibition of the autophagy pathway was protective. Necrosis follows autophagy in both polyp and colony assays. These results establish the hydroid allorecognition system as a novel model of cell death: one in which apoptosis is not elicited, but both autophagy and subsequent necrosis is directly elicited by an allorecognition event.

## Methods

### Ethics

Animal care at Yale University is conducted under the supervision of the Yale Animal Care and Use Committee. Hydroids are diploblastic organisms lacking a brain and are not governed by specialized guidelines. Animals are reared in recirculating aquaria using standard methods [32]. Animals sacrificed for electron microscopy were clonal explants of animals maintained in continuous culture and were narcotized prior to fixation as detailed below.

### Animals and Fusibility Assays

With one exception, all colonies were derived from established congenic lines. The derivation of these lines is available by concatenating pedigrees available elsewhere [26], [28], [33]. ARC genotype is designated as *alr1alr2*/*alr1alr2* and the letters *f* and *r* haplotype. Transitory fusion occurs when either *fr/ff* or *rf/ff* colonies are paired with *rr/rr* colonies. Unless otherwise stated, all observations were conducted using *rf/ff* versus *rr/rr* pairing. The identity of individual colonies is reported in Table S1. Details of animal husbandry are given elsewhere [32].

Two allorecognition assays were utilized. The ‘colony assay’ mimics a natural encounter. Small pieces of stolonal mat bearing three feeding polyps from each of the two colonies meant to interact are surgically removed from stock colonies and placed adjacent to one another, and affixed to a glass microscope slide or a cover slip using quilting thread. Within 1–3 days, colonies have adhered to the slide by new growth and the threads are removed. Observations begin when colonies grow into contact [8], [34], typically between three days and one week after having been established.

The colony assay has a particular virtue in the study of dynamical processes such as apoptosis or autophagy for which sampling at appropriate time-points can be essential. The colony assay obviates the temporal sampling issue. As two colonies grow the perimeter in contact expands. When the tissue is fixed at a point when the colonies are separating in the center of the contact zone, tissues that have been in contact for shorter intervals necessarily surround the interior region. By scanning the entire contact zones one can be confident that results are not an artifact of sampling.

The second assay is a grafting experiment [9], [10], [28], [35], with no natural analog. Polyps from colonies to be tested are severed at the base of the body column and both threaded onto a human hair with their bases tightly pressed together using agar blocks. After 2 hours, agar blocks are removed and the now adherent polyp graft gently removed from the hair. The graft is placed in a glass petri plate and observed daily.

### Imaging

#### *In vivo* time-lapse digital microscopy

A stage-mounted, flow-through culture system was developed for continuous digital microscopic imaging of a transitory fusion reaction from the time at which a colony encounters another until the colonies have completely separated. A Warner RC-50 flow-through chamber was modified to accept coverslips as top and bottom surfaces of a closed chamber. Colonies were grown on 22×40 mm cover slips, which served as the bottom surface of the chamber. The interior of the chamber was formed from a hexagonal 36×18 mm opening cut into a 6.1 mm plexiglass disk with an internal volume of 4 ml and opposing 16 gauge stainless steel inlet and output ports. Cover slips and chamber are sealed with vacuum grease, sandwiched by 0.38 mm silicone gaskets, and covered a 40 mm diameter 1.1 mm thick coverslip compressed onto the top of the stack by a threaded aluminum chamber top. Water was continuously exchanged by Harvard PHD 2000 screw feed push/pull infusion pump fitted with 4×50 ml syringes feeding a system of check valves to create continuous 5 ml/minute flow. Colonies were imaged using either a Zeiss Axiovert inverted compound microscope or a Zeiss Lumar dissecting microscope at the maximal magnification that permitted the contact area to be completely or, after initial growth, nearly completely imaged (ca. 25–50X). Images were acquired at 9 minute intervals. Imaging is interrupted every two days for up to 1.25 hours during which colonies were hand fed 3–4 day *Artemia salina*, without removing them from the chamber and the contact area cleaned with a fine camel hair brush if necessary. Five replicate movies were generated. For one film, the gastrovascular anatomy in the contact zone was traced for selected frames using a Wacom Cintiq 24D graphics monitor and a movie generated of the resulting schematics.

**Scanning electron microscopy.** Colonies were allowed to grow into contact on glass cover slips and fixed when a portion of the contact zone had separated while other regions remained fused. Colonies were relaxed in menthol, fixed using a solution of 4% gluturaldehyde and FSW (pH 8) for one hour, followed by three 5 minute rinses with double salinity, 0.22 µl filtered sea water (FSW), and post-fixed for one hour in 1% osmium tetroxide/FSW. After three washes with DI water, the colonies were dehydrated from DI water to 100% ethanol via five 10-minute washes in 70%, 80%, 90%, and two washes in 100% ethanol. Samples remained immersed in 100% ethanol until 100% dehydrated in a critical point drier, covered with gold in a EMS 550 sputter coater, and examined with a ISI SS40 scanning electron microscope equipped with Orion 32 imaging software.

#### Transmission electron microscopy

Observations were made on both *fr/ff* and *rf/ff* colonies paired with *rr/rr* colonies (Table S1), as well as isogenic control fusions. In each case, colonies were allowed to grow into contact on a substrate of Embed-812. Colonies were relaxed, fixed and dehydrated as described for SEM, followed by three 5 minute washes with propylene oxide and incubation for one hour in 1∶1 Embed-812-propylene oxide, 12 hours in 2∶1 Embed-812: propylene oxide, and 2 hours in pure Embed-812. Samples were then embedded for 12 hours in a vacuum oven. Tissue was sectioned using a MT-1 ultra-microtome and a Diatome diamond knife, and section thickness assessed by color, with all sections used for TEM being 600–750 Å (gold or silver). Sections were stained for 10 minutes in uranyl acetate, washed 10 times in DI water, stained for 5 minutes with lead citrate, and washed 10 times in DI water. All sections were viewed with a Zeiss EM-900, and imaged with a Mega View III Soft Imaging System. Isogeneic and allogeneic polyp grafts were treated in the same fashion, except that fixation was performed at pH 7.5 and stock animals included a wild-type colony known to undergo transitory fusion with the congenic line (Table S1).

### TUNEL Assays

TUNEL assays were performed both on polyp grafts (*rf/ff* versus *rr/rr*) and on colony assays (*fr/ff* versus *rr/rr*) grown on cover slips. Tissues fixed in 4% paraformaldehyde were washed in PBS for 5 minutes, permeabilized with proteinase K (20 µg/ml) for 20 minutes at room temperature, and then washed twice for 5 minutes in PBS. Positive controls were treated with DNAase and likewise washed twice for 5 minutes in PBS. Tissues were covered with 100 µl of Equilibrium Buffer (Promega Dead End Fluorometric TUNEL kit) for 10 minutes and blotted with 100 µl of freshly made TdT buffer reaction mix (nucleotide mixture, 50 µl; TdT enzyme, 10 µl; and Equilibrium Buffer, 450 µl), incubated for 60 minutes at 37°C in a dark, humidified chamber and the reaction terminated by immersion in 2X saline sodium citrate for 15 minutes in the dark. Samples were washed in 0.1% Triton X-100+5 mg/ml BSA in PBS for 5 minutes, then washed twice in PBS, followed by staining for propidium iodide (1 µg/ml in PBS) for 5 minutes in the dark, washed 3 times in PBS for 5 minutes, and mounted in 70% glycerol in PBS. Observations were made at 520 nm (FITC) for TUNEL staining and at >620 nm (DAPI) for propidium iodine staining.

### Pharmacological Experiments

We tested the effect on transitory fusion of treatment with the autophagy inhibitor 3-methyladenine (3-MA) and the autophagy activator rapamycin [36]. Interacting colonies were bathed in a solution of either 1 mM 3-MA or 1 µM rapamycin and assessed the time to initiation of separation in the continuous imaging colony assay [37]. If separation was not detected by 96 hours post-contact, the trial was terminated. We also investigated the effect of necrostatin, which disrupts the mammalian regulated necrosis pathway [38]–[40]. Necrostatin is costly and unstable, which led us to test its effect using polyp fusions, which can be maintained in small volumes. Six replicate allogeneic *fr/ff* versus *rr/rr* polyp grafts were generated and continuously incubated in either 15 ml of 30 µM necrostatin-1 in 0.2% DMSO or in 0.2% DMSO alone. Five additional isogeneic fusions were established and incubated in the same solutions. Necrostatin-1 and carrier fluids were exchanged twice daily. After 3 days, half of the necrostatin treated colonies were moved to a carrier alone treatment, while the remaining three replicates were maintained in the necrostatin treatment. Each graft was imaged daily using a Zeiss Lumar dissecting microscope.

## Results

### *In vivo* Time-lapse Microscopy

**Figure 1:**
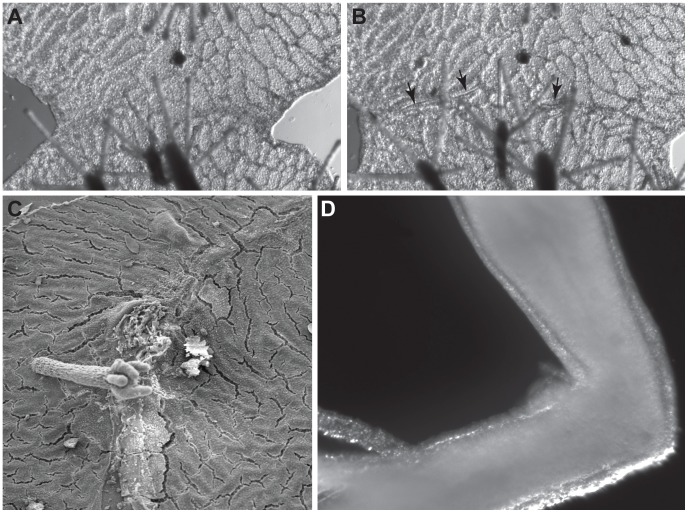
(A) Allogeneic colony fusion 22 hours post-contact (42X). (B) Same encounter as (A) at 55 hours post-contact. Arrowheads point to separation. (C) Scanning electron micrograph of colonies undergoing transitory fusion (80X). (D) Allogeneic polyp fusion showing necrotic cells shed at the graft boundary (108X).

Colonies fused within 9–18 minutes following initial contact. Thereafter gastrovascular transport in the contact zone coincided in timing and extent with that of internal regions of the two colonies within the field of view. Separation begins 46.3+/−4.6 hours (n = 5) following initial contact (Fig. 1A,B). Separation was nucleated at one to three locations along the contact zone. Initial separation occurred in the ectoderm of the stolonal mat in regions between endodermal gastrovascular canals that ran parallel to the contact zone and spread from that point. The rate of separation was heterogeneous, with ectodermal separation occurring at up to 0.7 µm/min, but with separation frequently stalled for up to several hours in locations where gastrovascular canals had once run normal to the fusion zone. The spatial distribution of gastrovascular canals remains unmodified in interacting colonies, except at the immediate locations (up to 25–50 µm) adjacent to where separation occurs. Separation does not always start where colonies initially came into contact, but may be displaced either laterally along the contact zone or in the direction of either of the two colonies. The maximum distance from separation point to an initial contact point was 103 µm. At no point were nematocytes seen to migrate to the contact zone. A movie of continuously imaged colonies undergoing transitory fusion and a schematic of gastrovascular architecture in selected frames are provided as Movie S1 and S2, respectively.

### Electron Microscopy

Scanning electron micrographs of the contact zone display a clear line of separation between two colonies, with considerable cellular debris. The region adjacent to the separated tissue was characterized by swelling of the ectoderm and a disruption of the glycocalyx (Fig. 1C).

**Figure 2:**
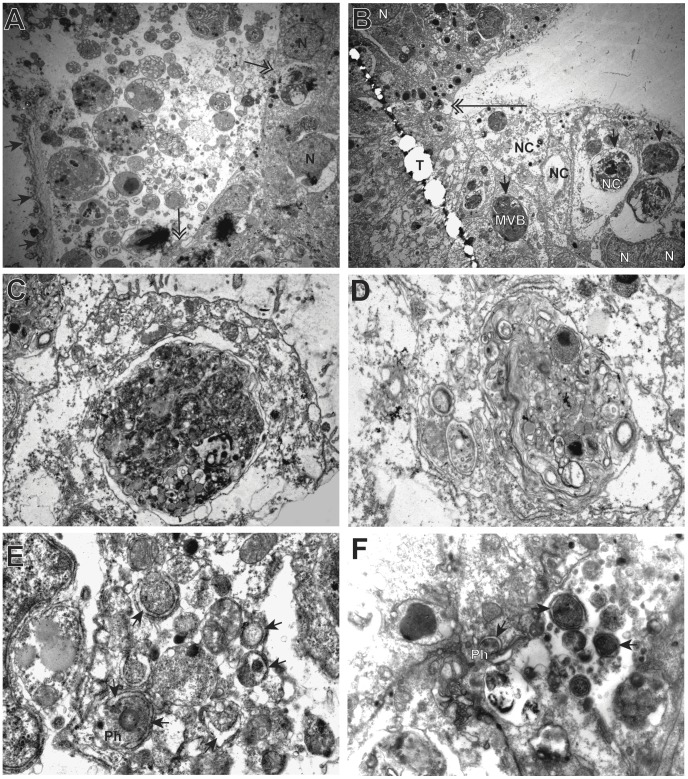
Transmission electron micrographs of allogeneic colony fusion. (A) Ectodermal margin of separated *fr/ff* versus *rr/rr* fusion zone after separation (3,000X). Arrows to glycocalyx, double-headed arrows to points of apparent loss of membrane integrity. (B) Contact zone between *rf/ff* versus *rr/rr* encounter (3,000X). Arrows point to multivestivular bodies (MVB), double-headed arrow designates point of contact. (C) MVB (12,000X). (D) Multilamellar body (MLB) (20,000X). Gastroderm of (E) *rf/ff* versus *rr/rr* encounter (20,000X) and of (F) *rf/ff* versus *rr/rr* (12,000X), showing numerous phagophores and autophagosomes (arrows). MVB, multivesticular body; N, nucleus; NC, necrotic cell; Ph, phagophores; T, tear in section.

Transmission electron micrographs from a region of separation from an encounter between a *rf/ff* and a *rr/rr* colony is shown in Fig. 2A. The glycocalyx was disrupted from the apical ectodermal surface and rent in some locations. Beneath the glycocalyx extensive cellular debris was evident, including many membrane bound structures, as well as light staining vacuolated ghosts. The cellular debris is coextensive with apical ectoderm in several locations. The apical ectoderm lacks the characteristic microvillar layer, and cells contain multivesticular bodies and multilamillar bodies. Nuclei are intact without blebbing of the nuclear membrane. Micrographs at the point of separation between a *fr/ff* and a *rr/rr* colony were similar (Fig. 2B). The ectoderm on both sides of the contact point lacks the characteristic microvillar layer. Ectoderm cell membranes were intact, but cells on one side of the contact were swollen with disrupted cytoplasm. Cells on either side of the contact point display multivesticular and multilamellar bodies of varying numbers and sizes (Fig. 2B,C,D). Nuclei are intact. Gastrodermal cells adjacent to the separated tissues were found to have disrupted apical cell membranes (Fig. 2E), with regions of intact cytoplasm characterized by numerous phagophores and autophagosomes (Fig. 2E, F).

**Figure 3:**
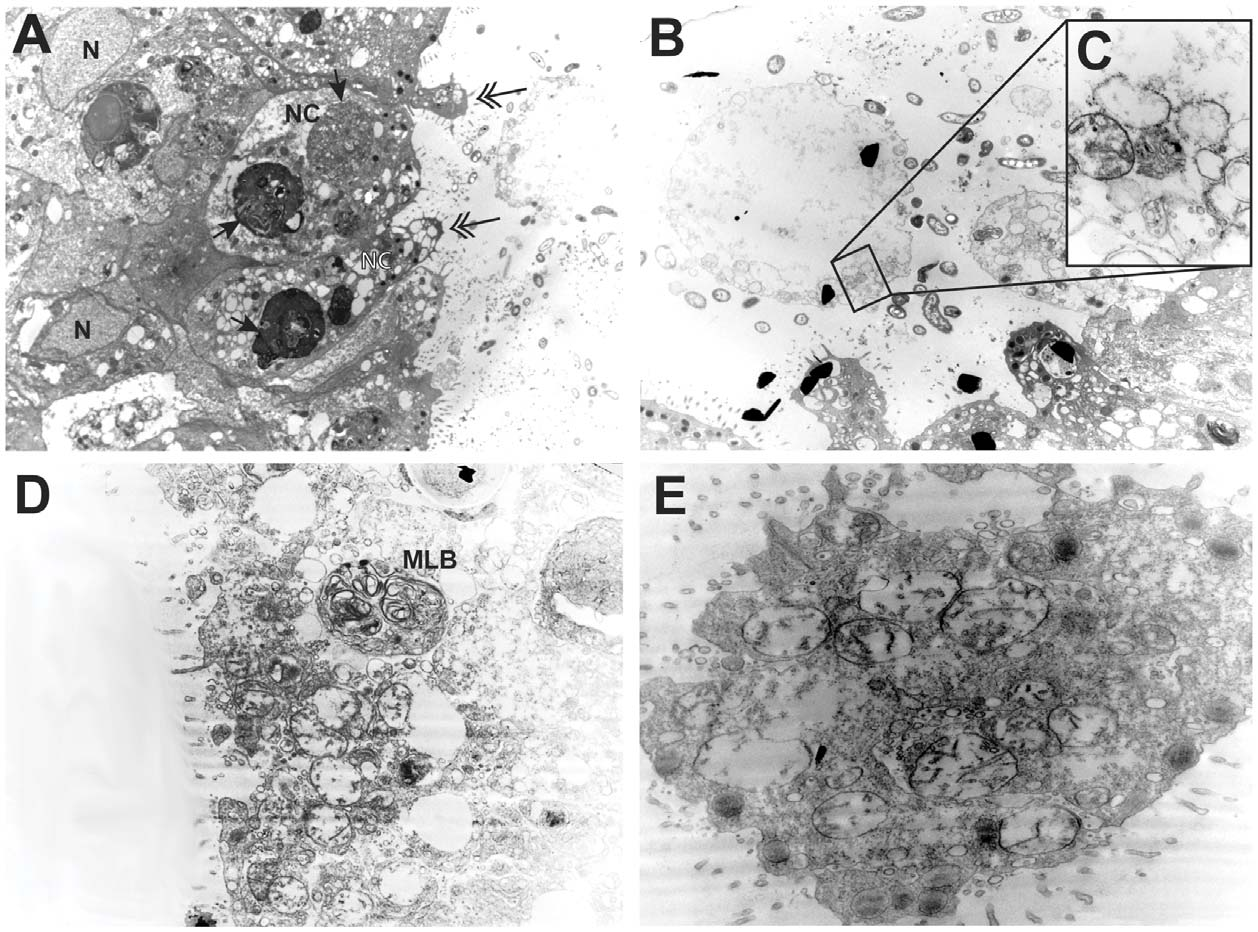
Transmission electron micrographs of allogeneic polyp fusions. (A) Ectoderm at graft margin (4400X) 24 hours post-fusion, arrows to multivesticular bodies, double-shafted arrows to sites of apparent loss of membrane integrity. (B) Ectodermal surface at graft margin 24 hours post-fusion showing vacuolar ghosts (20000X) including (C) mitochondria remnants (85000X). (D) Ectoderm at graft margin at 48 hours post-fusion (12000X). (E) Ectoderm at graft margin at 48 hours post-fusion (20000X). MLB, multilamellar body; N, nucleus; NC, necrotic cell.

In allogeneic polyp grafts, the region around the polyp graft was surrounded by a field of debris (Fig 1D). Micrographs of the ectoderm in this same region at 24 hours post-fusion were characterized by a lack of a microvillar layer and a substantial extracellular debris field (Figs. 3A, B), which appears coextensive with the ectoderm at some locations (Fig. 3A). Extracellular vacuolar ghosts (Fig 3B) were found to include mitochondrial remnants (Fig. 3C). Within the ectoderm, cells are highly vacuolated, densely populated by multivesticular bodies and several were found detached from the mesoglea (Fig. 3A). Nuclei of adjacent cells were intact, with no sign of nuclear membrane blebbing. At 48 hours, ectodermal cell membranes could no longer be detected (Fig. 3D), cellular material was fragmented and extensively vacuolated, and mitochondria were swollen with disordered cristae (Figure 3D,E). Multilamellar bodies (Fig. 3D) and multivesticular bodies were common.

**Figure 4:**
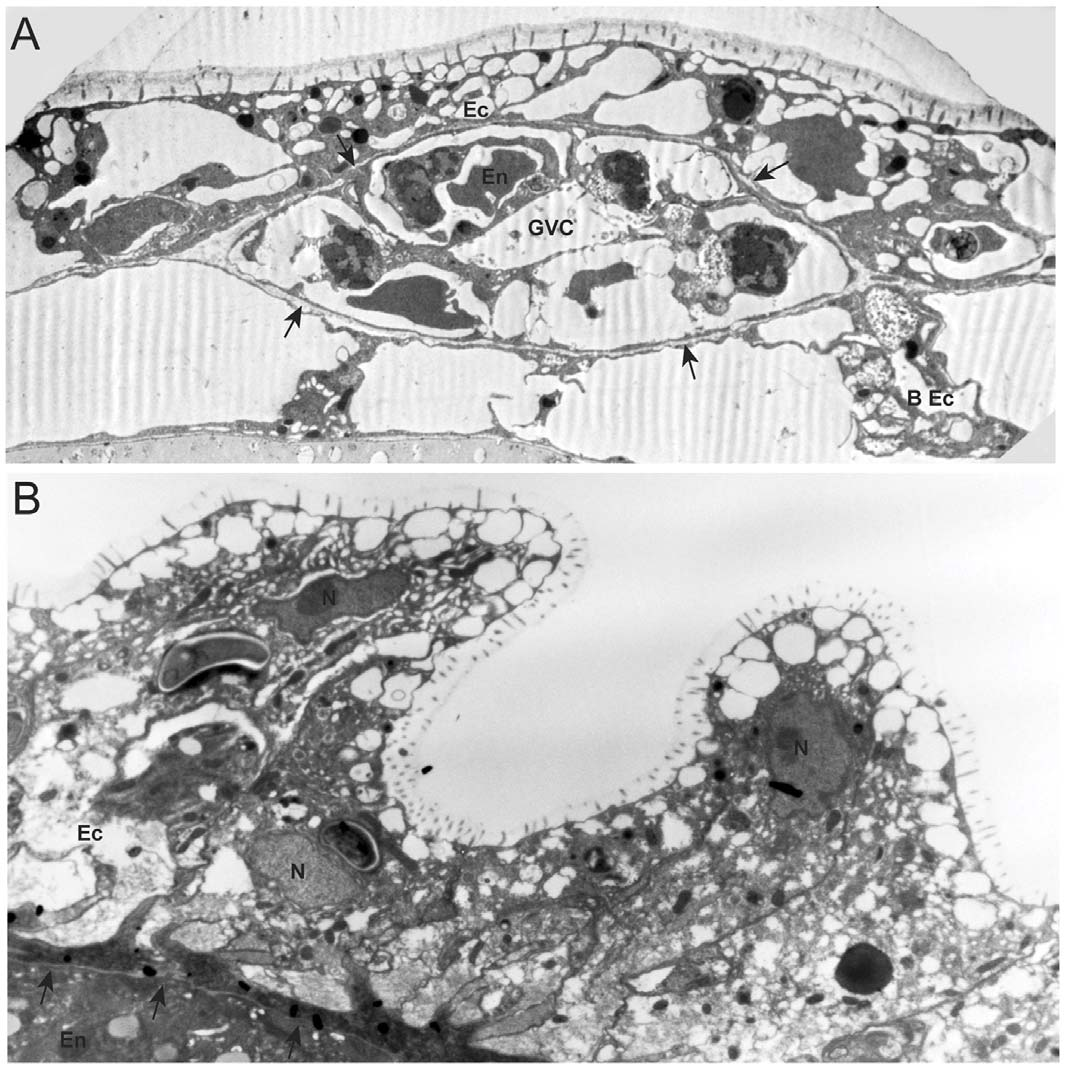
Transmission electron micrographs of isogeneic fusions. (A) Colony assay (3000X). (B) Polyp assay (4400X). Arrows point to mesoglea, BEc, basal ectoderm; Ec, ectoderm; En, endoderm; GVC, gastrovascular cavity, N, nucleus.

Micrographs of an isogeneic colony and polyp fusion are shown in Figure 4A and 4B respectively. The control isogeneic colony and polyp fusion display no autophagic or necrotic features.

### Pharmacological Manipulations

The autophagy pathway is regulated by the target-of-rapamycin (TOR) pathway [41], which can be manipulated pharmacologically. 3-methyladenine (3-MA) suppresses autophagophore formation by disruption of the class 3 phosphoinositide 3-kinase (PI3K) activity [42]. Treatment of interacting colonies with 3-MA was strongly protective. While the average time to separation for 5 control colonies was 46.3+/−4.6 hours (n = 6), all 5 replicates treated with 3-MA remained fused for the entire 96 hour treatment period. One colony was maintained in continuous culture with 3-MA for a further 56 hours and this single encounter had not separated when the trail was terminated after 152 hours in contact (movie S3). After removal of 3-MA, all colonies separated after 26.7+/−21.7 hours. Colonies treated with 3-MA grew into contact and developed normally.

Rapamycin upregulates autophagy via TOR [41]. While colonies treated with rapamycin separated earlier than control colonies (37.7+/−9.1, n = 4), this difference was not significant (t-test, p = 0.079).

Regulated necrosis is a mammalian cell death pathway elicited by association of Receptor Interacting Protein 1 (RIP1) and RIP3 in the cytosol [39], [43], [44]. Necrostatin-1 specifically inhibits RIP1 kinase and disrupts regulated necrosis in tissue culture [38]–[40]. Treatment of grafted polyps with necrostatin was strongly protective, but the reagent was observed to have profound effects on metabolism. All control isogenic colonies displayed a continuous gastrovascular cavity with no cellular debris surrounding the fusion zone at any time point (Fig. S1A). In half the replicates (n = 3), polyps and their tentacles shrank. Isogeneic grafts incubated in necrostatin displayed gastrovascular continuity, but neither shrank no shortened their tentacles (Fig. S1B).

After three days, control allogeneic colonies had shrunk and lost their tentacles. Half had separated (n = 3) and the remaining (n = 3) exhibited substantial cellular debris at the graft margin (Fig. S1C,D). In contrast, at three days, all six necrostatin treated allogeneic grafts were attached with clear tissue continuity and without cell debris at the graft junction (Fig. S1E). Necrostatin treated grafts removed from necrostatin exposure on day 3 (n = 3) lost their tentacles and bore cellular debris encircling the graft margin after 24 hours, while grafts retained in necrostatin (n = 3) remained fused with neither necrosis at the graft margin nor loss of tentacles. Necrostatin treated allogeneic grafts did not display the tentacle shortening observed in control allogeneic colonies, nor had the polyps shrunk in size. Tentacles were held rigidly upright, although the probing the tentacles with forceps elicited normal contraction behavior and tentacles responded normally when presented with an *Artemia* nauplius.

### TUNEL Assays

Signal was detected in DNAase controls, but no signal of DNA fragmentation was observed in either colony or grafted polyp assays (data not shown).

## Discussion

Three modes of regulated cell death are known: apoptosis, autophagy, and regulated necrosis. Apoptosis, often called Type I programmed cell death, is the default cell-death pathway in a number of systems and may be triggered by either binding of target molecules at the cell membrane or by mitochondrial activation [45], [46]. Autophagy, sometimes referred to as Type II programmed cell death, is better known as a cell protective process with a role in nutrient starvation [41]. When nutrients are restricted, cells elaborate double-walled membranes known as phagophores, which enclose cell constituents to form autophagosomes that subsequently fuse with lysosomes to produce autophagolysosomes. Regulated necrosis, also called Type III programmed cell death, or necroptosis [39], [43], [44], [47]–[49], is known only from mammals, is sometimes associated with autophagy and is often elicited only if the apoptotic cell death pathway is disrupted. Our results suggest that autophagy and necrosis are involved in the colony separation process of a transitory fusion allorecognition encounter, while apoptosis is not.

Our observations provide no evidence for apoptotic cell death in the transitory fusion phenomenon. The diagnostic ultrastructural signatures of apoptosis include blebbing of the nuclear membrane and sharply delineated masses of condensed chromatin at the nuclear periphery [50]. Apoptosis is accompanied by DNA fragmentation [51]. Apoptosis has been reported in hydroids [52], including *Hydractinia echinata* [53]. Here, we have searched for evidence of apoptosis during transitory fusion. Nuclei are intact in all stages of transitory fusion observed, even in cells undergoing apparent loss of cell membrane integrity. Attempts to detect nuclear fragmentation by TUNEL showed that DNA fragmentation does not accompany the transitory fusion cell death. Our observations provide no evidence for apoptosis cell death in the transitory fusion phenomenon.

We observed widespread autophagy in the transitory fusion zone of colony assays, with phagophores and autophagosomes detected in both intact and necrotic cells. Both phagophores and autophagosomes bear a distinctive ultrastructural signature [54]. Autophagolysosomes frequently appear as multivesticular (MVB) or multilamellar (MLB) bodies. MVB’s and MLB’s were widespread in both the ectoderm and the endoderm of colony assays. In polyp fusions, where only the ectoderm was observed, both MVB’s and MLB’s were observed at high frequencies. No signatures of autophagy were found in the ultrastructure of isogeneic fusions or in regions of colony or polyp grafts in excess of 10 cell diameters from the fusion zone. No differences were detected between polyp fusions and colony assays, nor were differences detected in whether the mismatch in allodeterminants was in *alr1* or *alr2*.

Autophagic progression may be altered pharmacologically via disruption of the nutrient sensor target-of-rapamycin (TOR) [42], [55], [56]. TOR acts downstream of the class 3 phosphoinositide 3-kinase (PI3K) and 3-methyladenine (3-MA) suppresses autophagophore formation by disruption of the class 3 phosphoinositide 3-kinase (PI3K) activity. Rapamycin, conversely, acts as an inducer of autophagy by inhibiting TOR activity [55]. The genome of the hydroid *Hydra* contains all key elements of this pathway [57]. We found that incubation in 3-MA masked transitory fusion *in vivo*. Transitory fusion was restored only when exposure to the reagent was terminated, after which transitory fusion occurred at an accelerated pace as expected of a control process. Rapamycin had no effect in the same assay.

3-MA is a non-specific PI3K inhibitor, influencing not only the class 3 PI3K interactions required for autophagy, but also class 1 PI3K activity [37], [41], [58]. Concern over non-specific effects are mitigated in our experiments by the fact that 3-MA is strongly protective when colonies are exposed to concentrations an order of magnitude lower that that used in conventional tissue culture studies [37]. Also reassuring is the fact that the assay utilized was performed *in vivo* and the continuous digital imaging show that the animals continued to grow, feed, and circulate gastrovascular fluids normally throughout the experiment. Nonetheless, it would be desirable to supplement the pharmacological studies reported here with transgenic experiments, newly available for *Hydractinia* [59], designed to disrupt key autophagy-related genes (*atg*).

The autophagic response we observe might be a response to nutrient deprivation in the contact zone. Several facts suggest otherwise. First, gastrovascular flow to the colony periphery continues throughout the process as evident in time-lapse imaging (movies S1, S3). Second, vascular remodeling takes place quickly, immediately preceding separation (movie S1, S2, S3) at a time during which autophagy is already well advanced. Moreover, studies of nutrient deprivation in *Hydra* have shown that well-fed animals must be starved for 10 days to induce autophagy [57]. Yet our experiments show that colonies have completed separation in two days or less. Together these findings imply that allodeterminant incompatibility recruits the autophagic pathway.

The lack of an effect of rampmycin treatment may reflect the dosages we utilized. Studies of autophagy in starved *Hydra* detected an effect at dosages an order of magnitude larger than the conventional dosage we utilized [57]. A more intriguing alternative is that the pathway from allorecognition receptor binding to the induction of autophagy may not include TOR.

Necrosis has long been attributed to a catastrophic unregulated form of cell death associated with swelling of the cell and loss of membrane integrity, without either the involvement of the lysosomes characteristic of autophagy or the nuclear blebbing and DNA fragmentation characteristic of apoptosis. The mammalian regulated necrosis pathway is dependent upon the formation of a complex between two serine threonine kinases, receptor-interacting protein 1 (RIP1) and RIP3 [39], [43], [44]. Necrostatin specifically inhibits RIP1 and is strongly protective in tissue culture [40]. We have found that treatment of polyp grafts with necrostatin masked the transitory fusion response. Interpretation of this striking response is not straightforward. A survey of the two cnidarian genomes now available, the hydroid *Hydra* and the anthozoan *Nematostella*, reveal no clear cnidarian orthologs of either RIP1 or RIP3. The binding partner to necrostatin in *Hydractinia* is unknown. Furthermore, the drug appeared to have a strong effect on overall metabolism, in that polyps and tentacles shrank in both isogenic and allogeneic controls, whereas necrostatin polyps neither shrank nor resorbed their tentacles. Necrostatin, then, may be acting in this hydroid as a potent disruptor of metabolic activity and the pronounced protective effect in allografts may simply reflect the fact that cell death is an active metabolic process. Further study of the necrostatin’s effect on hydroids is warranted in either instance. Should necrostatin’s protective effect on allorecognition be a side effect of the drug’s effect on metabolism, the observed necrosis may be an unregulated process secondary to the extensive autophagy or a form of regulated necrosis distinct from mammalian necroptosis.

In mammalian systems, unambiguous evidence that autophagy contributes to cell death independent of apoptotic or regulated necrosis pathways is largely lacking [60]. Rather, autophagy in mammalian cell death is typically assigned the quixotic role of a failed attempt at cell protection. Yet autophagy has additional roles, notably in immunity and in development [41], [61]. In non-mammalian systems, developmental cell death is often associated with autophagy and subsequent necrosis. In *Drosophila*, salivary cells die at a specific point in development and the cell death is characterized by apoptosis and autophagy. Elimination of salivary glands is inhibited in *atg* mutant flies [62]. In *Caenorhabditis*, autophagy is required for ionic imbalance-induced necrotic neuronal cell death [63]–[65]. In the cellular slime mold *Dictyostelium*, which does not display apoptosis, autophagy is required for multicellular development [66]–[68].

Autophagy also plays an important role in the processing of immunoregulatory molecules and in both the control of and failure to control intracellular invaders [61]. Notably, though, a role for autophagy in graft rejection has not previously been reported. Although histological studies of failed grafts display widespread autophagy and necrosis, such responses may well be explained as sequelae of graft ischemia that occurs during surgery. The principle line of defense against allografts in mammals is the adaptive immune system. This fact, however, does not imply that a more primitive innate mechanism is absent [69], [70]. If such a primitive mechanism exists and if it were to share ancestry with the allorecognition system of hydroids, one might expect contact between mammalian allogeneic somatic cells to elicit autophagy and necrosis. Curiously, experiments involving non-immune allogeneic somatic cell lines do not seem to have been reported. Moreover, the association of allorecognition and autophagy is not without precedent. Recent work has shown that, following fertilization, male mitochondria are eliminated by autophagy in both *Caenorhabditis* [71] and mice [72].

This study is the first to show that cell-cell contact between allogeneic tissues can elicit autophagy and necrosis. Moreover, we showed that pharmacological disruption of the autophagy pathway completely suppresses the transitory fusion phenotype. These findings support the earlier hypothesis that the nematocyst-based effector system is derived and establish the *Hydractinia* allorecognition system as a unique model for the study of cell death.

## Supporting Information

Figure S1 **Polyp grafting experiments.** (A) Isogeneic (*rr/rr* versus *rr/rr*) control grafts. (B) Isogeneic grafts incubated in necrostatin. (C) Allogeneic (*fr/ff* versus *rr/rr*) control grafts. (D) Close-up of graft margin in an allogeneic control showing cellular debris. (E) Allogeneic grafts in necrostatin. Scale: 200 um.
(TIFF)

Table S1 **Colony identifications.**
(DOCX)

Movie S1 **Continuous time-lapse imaging of** ***rf/ff*** **versus** ***rr/rr*** **transitory fusion reaction at 9-minute intervals (46X).** Colonies began to separate at 52∶12 hours following initial contact.
(MP4)

Movie S2 **Movie generated from tracings of gastrovascular canals of selected frames of Movie S1.** Frames were chosen to minimize the extent to which polyps obscure the fusion zone. The time post-contact is given on each frame (hours:minutes). Note that gastrovascular architecture in the fusion zone is largely constant once initiated and that gastrovascular remodeling associated with separation is local and occurs immediately prior to separation. See also movie S1, S3.
(MP4)

Movie S3 **Transitory fusion reaction of an** ***rf/ff*** **versus** ***rr/rr*** **while incubated in 3-MA (38X).** To minimize file size the film begins 67∶40 post-contact. Frames acquired after removal from 3-MA at 152∶00 hours are designated.
(MP4)
