# Supplementary figures and images for: Allorecognition Triggers Autophagy and Subsequent Necrosis in the Cnidarian Hydractinia symbiolongicarpus
Source: PLoS One. 2012 Nov 8;7(11):e48914. doi: 10.1371/journal.pone.0048914 (PMC3493586; doi:10.1371/journal.pone.0048914)

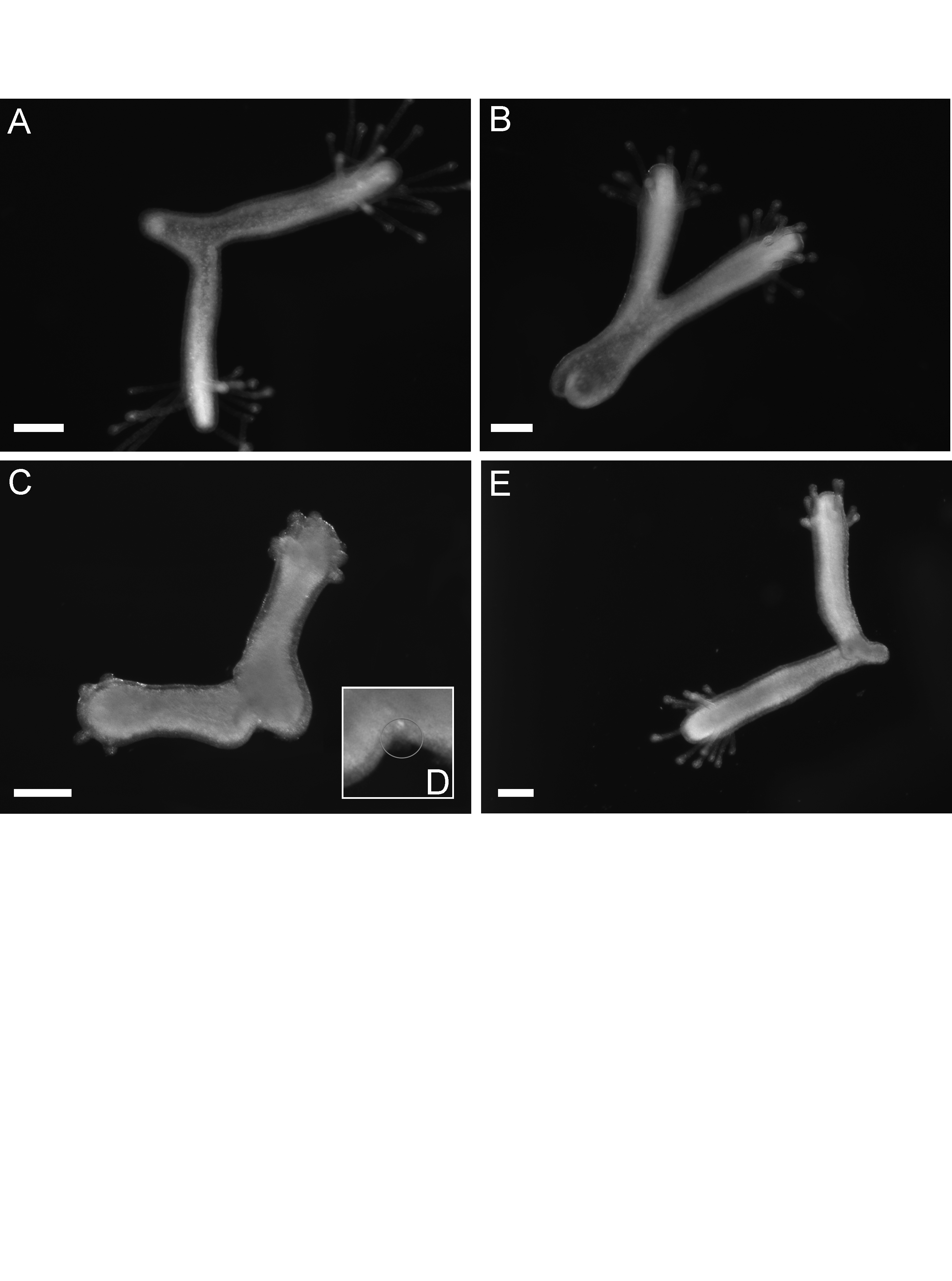

Supplement: Figure S1 — Polyp grafting experiments. (A) Isogeneic (rr/rr versus rr/rr) control grafts. (B) Isogeneic grafts incubated in necrostatin. (C) Allogeneic (fr/ff versus rr/rr) control grafts. (D) Close-up of graft margin in an allogeneic control showing cellular debris. (E) Allogeneic grafts in necrostatin. Scale: 200 um. (TIFF) [file pone.0048914.s001.tiff]
