# Supplementary material for: Allorecognition Triggers Autophagy and Subsequent Necrosis in the Cnidarian Hydractinia symbiolongicarpus
Source: PLoS One. 2012 Nov 8;7(11):e48914. doi: 10.1371/journal.pone.0048914 (PMC3493586; doi:10.1371/journal.pone.0048914)
Supplement: Table S1 — Colony identifications. (DOCX) [file pone.0048914.s002.docx]

Supplementary Table 1. Colony identifications

| Colony ID | ARC | Colony ID | ARC | Assay | Observation |
| --- | --- | --- | --- | --- | --- |
| AP100-1747 | *rf/ff* | MP104-34 | *rr/rr* | Colony | SEM, TEM |
| AP100-451 | *fr/ff* | MP104-34 | *rr/rr* | Colony | TEM |
| AP100-1747 | *rf/ff* | MP104-34 | *rr/rr* | Colony | Time-lapse imaging |
| BC1-379 | *fr/ff* | 4117-2 | *rr/rr* | Colony | TUNEL |
| BC1-81 | *rf/ff* | 4117-2 | *rr/rr* | Polyp | TUNEL |
| V3 | *cc/ff* | LH06-082 | *f2f2/bb** | Polyp | TEM |
| LB 235-13 | *fr/ff* | LB245-7 | *rr/rr* | Polyp | Necrostatin experiments |

* Wild-type known to display transitory fusion reaction due to an F-like allele (F2) at *alr1 and alr2* [[1](#_ENREF_1),[2](#_ENREF_2)]

1. Nicotra ML, Powell AE, Rosengarten RD, Moreno M, Grimwood J, et al. (2009) A hypervariable invertebrate allodeterminant. Current Biology 19: 1-7.

2. Rosa SFP, Powell AE, Moreno M, Grimwood J, Lakkis FG, et al. (2010) *Hydractinia* allodeterminant *alr1* resides in an invertebrate immunoglobulin superfamily-like gene complex. Current Biology 20: 1122-1127.
